# Differences in association of lower bone mineral density with higher coronary calcification in female and male end-stage renal disease patients

**DOI:** 10.1186/s12882-019-1235-z

**Published:** 2019-02-18

**Authors:** Zhimin Chen, Abdul Rashid Qureshi, Torkel B. Brismar, Jonaz Ripsweden, Mathias Haarhaus, Peter Barany, Olof Heimburger, Bengt Lindholm, Peter Stenvinkel

**Affiliations:** 1 Kidney Disease Center, The First Affiliated Hospital, College of Medicine, Zhejiang University, Hangzhou, China; 2 Division of Renal Medicine and Baxter Novum, Department of Clinical Sciences, Intervention and Technology, Karolinska Institutet, Stockholm, Sweden; 3 Division of Medical Imaging and Technology, Department of Clinical Science, Intervention and Technology, Karolinska Institutet, and Department of Radiology, Karolinska University Hospital, Huddinge, Stockholm, Sweden

**Keywords:** Bone mineral density, Coronary calcification, End-stage renal disease

## Abstract

**Background:**

Risk of cardiac events and cardiovascular disease (CVD) in end-stage renal disease (ESRD) patients are predicted by coronary artery calcification (CAC) independently. It is not clear to what extent low bone mineral density (BMD) is associated with higher risk of CAC and if sex interacts. We investigated the sex-specific associations of CAC score with total body BMD (tBMD) as well as with BMD of different skeletal sub-regions.

**Methods:**

In 174 ESRD patients, median age 57 (10th–90th percentiles 29–75) years, 63% males, BMD (measured by dual-energy X-ray absorptiometry; DXA), CAC score (measured by cardiac CT) and circulating inflammatory biomarkers were analysed.

**Results:**

A total of 104 (60%) patients with CAC > 100 AUs were older, had higher prevalence of both clinical CVD and diabetes, higher level of high sensitivity C-reactive protein, tumour necrosis factor, interleukin-6 and lower T-score of tBMD. Female patients had significantly lower tBMD and BMD of all skeletal sub-regions, except head, than male patients. Female patients with high CAC (> 100 AUs) had significantly decreased T-score of tBMD, and lower BMD of arms, legs than those low CAC (≤ 100 AUs); elevated CAC score were associated with tBMD, T-score, Z-score of tBMD and BMD of arms and legs, while no such differences was observed in males. Multivariate generalized linear model (GLM) analysis adjusted for age, diabetes and hsCRP showed that in females per SD higher CAC score (1057 AUs) was predicted by either per SD (0.13 g/cm^2^) lower tBMD or per SD (0.17 g/cm^2^) lower BMD at legs. No such associations were found in male ESRD patients.

**Conclusions:**

In female, but not male, lower BMD, in particular sub-regions of legs, was associated with higher CAC score independently. Low BMD has the potential to identify increased risk for high CAC score in ESRD patients.

**Electronic supplementary material:**

The online version of this article (10.1186/s12882-019-1235-z) contains supplementary material, which is available to authorized users.

## Background

Cardiovascular disease (CVD) is a main cause of morbidity and mortality of end-stage renal disease (ESRD) patients [1] and the risk of CVD is predicted by coronary artery calcification (CAC) independently in ESRD patients [2, 3]. Thus, CAC can be an independent risk factor of CVD beyond conventional risk factors [4, 5]. In ESRD patients, chronic kidney disease (CKD) - mineral and bone disorders (CKD-MBD) is a major complication. Decreased bone mineral density (BMD) associates with increased fracture risk and predicts higher mortality and cardiovascular events in CKD patients and the general population [6–8]. Low BMD is associated with higher risk of CVD [6, 9, 10].

Bone mineralization and vascular calcification share some common pathways [11, 12]. An association of reduced BMD with vascular calcification has been found in the general population [13, 14] as well as in ESRD patients [15, 16]. However, no such association was found in some other studies in the general population [17–20] and ESRD patients [21]. Decreased cortical bone density has been found to associate with CAC [22], and progression of CAC was predicted by osteoporosis [23] in dialysis patients. Several molecular mechanisms have been suggested for the link between bone metabolism and vascular calcification [24].

Since it is not clear to what extent a decreased BMD may be linked to increased risk of CAC and other manifestations in ESRD patients, we investigated sex-specific associations between total body BMD (tBMD) and BMD of different skeletal sub-regions, determined by dual-energy X-ray absorptiometry (DXA), and CAC, determined by computed tomography (CT) of the heart.

## Methods

### Patients

One hundred seventy-four ESRD patients with median age of 57 (10th–90th percentiles 29–75) years, 63% males were enlisted at the Department of Renal Medicine at Karolinska University Hospital at Huddinge, Stockholm, between March 2008 and June 2015. All patients from three different cohorts, who had undergone both coronary CT and DXA measurements, were included, 69 (40%) were incident dialysis patients, 67 (38%) prevalent peritoneal dialysis (PD) patients and 38 (22%) recipients of living donor kidney transplant (LD-Rtx). The Ethics Committee of Karolinska University Hospital Huddinge approved the study protocols. Informed consent in written was obtained from all patients. Baseline characteristics of the 174 included ESRD patients are outlined in Table 1.

**Table 1:** Clinical and biochemical characteristics for the total 174 ESRD patients and for two subgroups based according to CAC score

	Total patients *n* = 174	Low CAC (≤ 100 AUs) (*n* = 70)	High CAC (>100 AUs) (*n* = 104)	*P* value
Demography and metabolic biomarkers				
Age, years	57 (29, 75)	41 (23, 63)	64 (49, 78)	< 0.001
Male, %	63	57	67	0.173
Diabetes, %	28	10	40	< 0.001
CVD, %	22	3	36	< 0.001
Body mass index, kg/m^2^	24.8 (19.9, 30.8)	23.9 (19.7, 30.7)	25.2 (20.7, 30.8)	0.080
Systolic BP, mmHg	139 (114, 169)	135 (114, 163)	143 (113, 179)	0.024
Diastolic BP, mmHg	83 (67, 97)	85 (70, 100)	81 (66, 96)	0.123
Hemoglobin, g/L	114 (94, 130)	113 (92, 128)	114 (100, 130)	0.064
Triglycerides, mmol/L	1.6 (0.9, 3.0)	1.6 (0.9, 2.8)	1.4 (0.9, 3.6)	0.039
Cholesterol, mmol/L	4.7 (3.3, 6.6)	4.6 (3.4, 6.1)	4.6 (3.0, 5.7)	0.175
HDL-cholesterol, mmol/L	1.2 (0.8, 2.3)	1.4 (0.9, 2.4)	1.1 (0.9, 2.3)	0.373
Creatinine, μmol/L	727 (491, 1012)	738 (509, 1185)	729 (414, 981)	0.014
S-albumin, g/L	33 (26, 39)	35 (28, 40)	32 (23, 38)	0.001
hsCRP, mg/L	2.1 (0.4, 18.7)	1.2 (0.2, 9.1)	3.6 (0.8, 35.4)	0.003
TNF, pg/ml ^a^	15.3 (9.9, 20.4)	12.9 (8.0, 19.8)	16.7 (12.0, 20.7)	0.003
IL-6, pg/ml ^b^	4.5 (0.9, 14.9)	2.1 (0.4, 8.1)	6.9 (2.2, 20.5)	< 0.001
PTX3 ng/mL ^c^	1.7 (0.7, 6.2)	1.8 (0.6, 7.1)	1.7 (0.7, 7.1)	0.758
Total testosterone in male, nmol/L^d^	11.0 (5.8, 20.5)	12.2 (7.4, 25.6)	10.2 (4.7, 16.9)	0.025
Mineral bone disease biomarkers				
iPTH, ng/L	300 (96, 655)	265 (113, 658)	320 (79, 735)	0.595
Calcium, mmol/L	2.3 (2.0, 2.5)	2.3 (2.1, 2.5)	2.2 (1.9, 2.6)	0.924
Phosphate, mmol/L	1.8 (1.2, 2.5)	1.9 (1.2, 2.4)	1.8 (1.2, 2.6)	0.559
1,25-OH vitamin D, nmol/L ^e^	13 (9, 28)	16.5 (9, 34)	11 (9, 23)	0.054
25-OH vitamin D, ng/L ^f^	29 (13, 70)	33 (14, 57)	29 (14, 80)	0.861
tBMD, g/cm^2^	1.12 (0.92, 1.30)	1.13 (1.00, 1.31)	1.10 (0.91, 1.27)	0.095
T-score of tBMD	−0.8 (−2.7, 1.1)	− 0.6 (− 2.0, 1.2)	−1.1 (− 3.1, 1.1)	0.006
Z-score of tBMD	− 0.4 (− 1.8, 1.2)	− 0.1 (− 1.5, 1.1)	− 0.5 (− 2.4, 1.3)	0.097

Data presented as median (range of 10th - 90th percentile) or percentage

Abbreviations: *BP* blood pressure, *HDL* high-density lipoprotein, *hsCRP* high sensitivity C-reactive protein, *TNF* tumor necrosis factor, *IL-6* interleukin-6, *PTX* pentraxin, *iPTH* intact parathyroid hormone, *CAC* coronary artery calcification, *tBMD* total bone mineral density

^a^*n* = 151, ^b^*n* = 166, ^c^*n* = 135; ^d^*n* = 95; ^e^*n* = 130, ^f^*n* = 105

### Measurements of BMD

BMD was measured by dual-energy X-ray absorptiometry (DXA) [25] and was presented in g/cm^2^ or T-score (i.e. the number of standard deviations difference in BMD compared to young adults of the same gender) or Z-score (indicating the number of standard deviations difference in BMD compared to an age-matched reference population of the same gender)). DXA was performed in all 174 patients using a DPX-L device (GE Lunar iDXA with software enCore 2008 version 12, 30, 008, GE Medical systems, Chalfont St. Giles, UK). BMD of total body (tBMD) and several skeletal sub-regions were also obtained: head, arms, legs, trunk, ribs, spine and pelvis.

### Measurements of CAC

CAC was measured by CT, a non-invasive approach, performed on a 64-channel detector scanner (LightSpeed VCT; General Electric (GE) Healthcare, Milwaukee, WI, USA) in cine mode. CAC was quantified in Agatston units (AUs) as a lesion with an area > 1 mm^2^ and a peak intensity > 130 Hounsfield Units (HUs) based on the Agatston method previously described in detail [26]. Details of CAC scan ascertainment and quantification have been published [27–29] in our previous study. In this study, we used a CAC score > 100 AUs which was associated with an higer risk of myocardial ischaemia and cardiac events [30] to identify patients with definite to extensive plaque burden.

### Laboratory analysis and other measurements

Plasma blood samples were received after an overnight fast in the morning in the ESRD patients. If not analysed immediately, the samples were kept frozen at − 70 °C. Plasma tumour necrosis factor (TNF), interleukin-6 (IL-6) and total testosterone concentrations were tested by commercial kits according to the instructions of the manufacturer available for an Immulite Automatic Analyzer (Siemens Medical Solutions, Los Angeles, CA, USA). Pentraxin-3 (PTX3) was tested by ELISA kits of R&D systems (Abingdon, UK). The level of haemoglobin, serum creatinine, triglycerides, cholesterol, and high density lipoprotein (HDL)-cholesterol, calcium, phosphorus, intact parathyroid hormone (iPTH), 25(OH) and 1,25(OH)_2_ vitamin D_3_, and high-sensitivity C-reactive protein (hsCRP) were tested by routine methods at the Department of Laboratory Medicine, Karolinska University Hospital, Huddinge, Sweden.

At the baseline body mass index (BMI) was recorded according to height and body weight. Arterial systolic and diastolic blood pressures (BP) were measured three times after a 15-min resting period in the morning. Earlier or present occurrence of documented of cerebrovascular, cardiovascular, or peripheral vascular disease like patients had suffered from cerebrovascular disease (stroke), myocardial infarctions, clinical signs of ischemic heart disease (angina pectoris), peripheral ischemic atherosclerotic vascular disease, had a history of an aortic aneurysm, mitral stenosis, and cardiac failure, considered as signs of CVD and details of CVD event determination have been published [31].

### Statistical analysis

Data are presented as median (range of 10th to 90th percentile) or percentage, as appropriate. Comparisons between two groups were assessed by the non-parametric Wilcoxon test for continuous variables and Fischer’s exact test for categorical variables. Spearman rank correlation analysis was used to determine associations between selected parameters. A receiver operating characteristics (ROC) curve was plotted for T-score of tBMD, age and hsCRP in relation to presence of high CAC score (***>***100 AUs). To study the associations between BMD, CAC score and other parameters, a multivariable generalized linear model (GLM) analysis were performed. In GLM analysis (stratified by gender), age, diabetes and hsCRP were included in the model. Statistical analyses were performed using statistical software SAS version 9.4 (SAS Campus Drive, Cary, NC, USA). Statistical significance was set at the level of *p* < 0.05.

## Results

### Clinical and biochemical characteristics of these ESRD patients

**Fig. 1:**
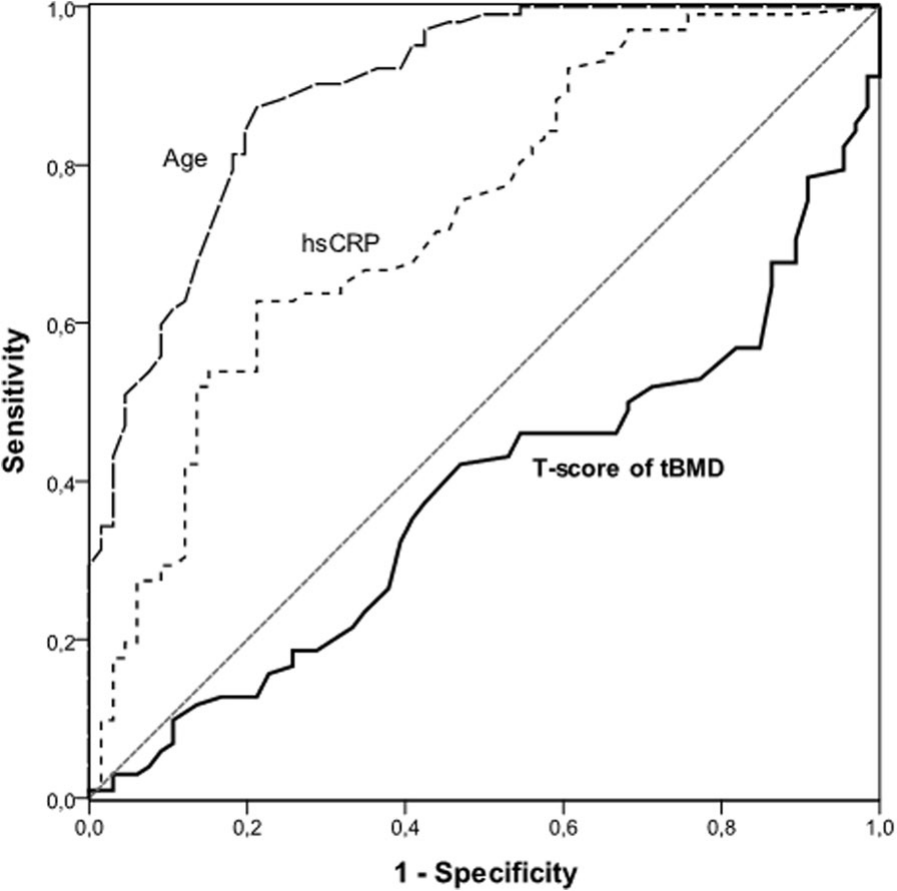
Areas under the curves (AUC) of receiver-operating characteristics curve (ROC) analysis for T-score of tBMD, age and hsCRP in relation to presence of high CAC score (>100 AUs). The separate AUCs are as follows: AUC of age (0.89), hsCRP (0.74) and T-score of tBMD (0.38)

Demographics and clinical characteristics of 174 ESRD patients are shown in Table 1; A total of 104 (60%) patients had CAC > 100 AUs. As expected, patients with high CAC (> 100 AUs) were older, higher prevalence of clinical CVD and diabetes, had higher levels of systolic BP, hsCRP, TNF, IL-6 and, lower levels of triglycerides, creatinine, serum albumin, total testosterone in male,and T-score of tBMD (Table 1). In receiver-operator characteristics curve (ROC) analysis, high CAC (> 100 AUs) was associated with age (AUC, 0.89), hsCRP (AUC, 0.74) and T-score of tBMD (0.38) (Fig. 1). When placed into two subgroups based on the median of tBMD level, are shown in Additional file 1: Table S1. Patients with tBMD lower than median (≤1.117 g/cm^2^; *n* = 87) had lower prevalence of male, lower BMI, creatinine, phosphate and higher concentration of HDL than those with tBMD higher than median (> 1.117 g/cm^2^; n = 87). No statistically significant differences were observed in any of the other variables (Additional file 1: Table S1).

### Differences of BMD in female and male ESRD patients

**Fig. 2:**
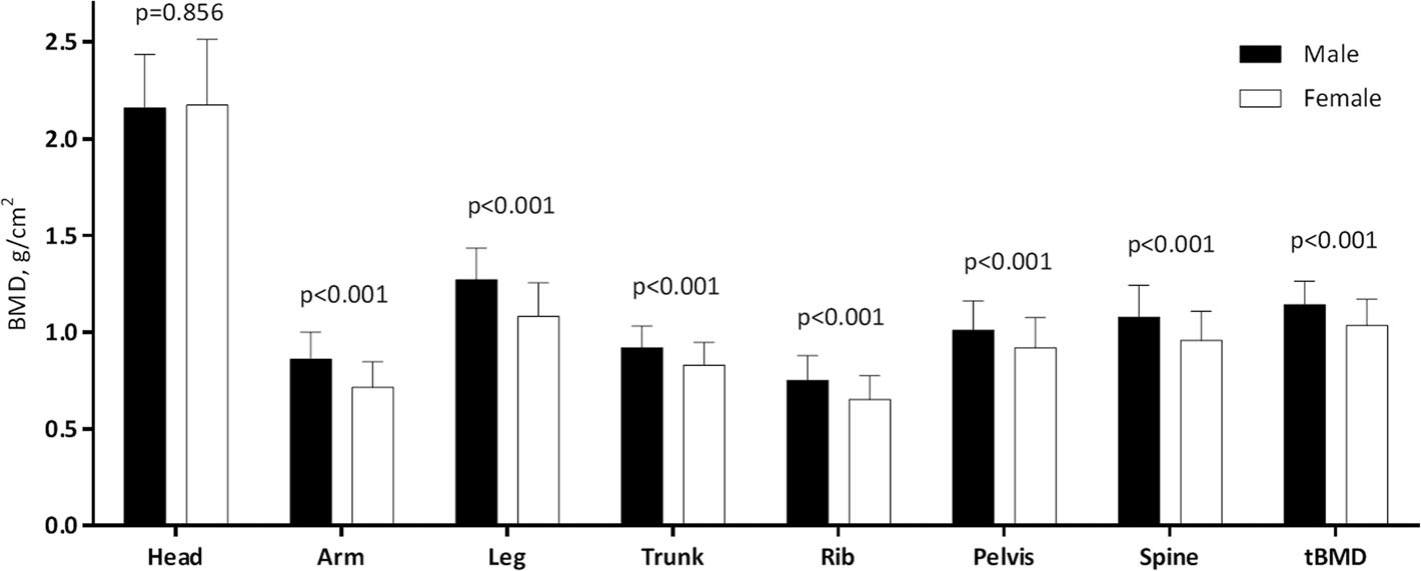
Differences of BMD at sub-regions and total BMD (tBMD) in male and female ESRD patients

Female ESRD patients had significantly lower tBMD and BMD of all skeletal sub-regions, except head, than male patients (Fig. 2). Female patients with high CAC (> 100 AUs) had significantly decreased T-score of tBMD and sub-regions BMD including armsand legs than patients with low CAC (≤ 100 AUs) (Table 2). No such differences were observed in males (Table 2).

**Table 2:** BMD at sub-regions and total BMD (tBMD) in low and high CAC groups stratified by gender

	Female			Male		
	Low CAC (≤ 100 AUs) (*n* = 30)	High CAC (>100 AUs) (*n* = 34)	*p*-value	Low CAC (≤ 100 AUs) (*n* = 40)	High CAC (>100AUs) (n = 70)	p-value
Head, g/cm^2^	2.19 (1.94, 2.65)	2.11 (1.73, 2.66)	0.162	2.25 (1.90, 2.60)	2.13 (1.73, 2.45)	0.023
Arms, g/cm^2^	0.73 (0.63, 1.01)	0.68 (0.49, 0.82)	0.029	0.86 (0.71, 1.09)	0.84 (0.67, 1.01)	0.233
Legs, g/cm^2^	1.16 (0.95, 1.30)	1.02 (0.78, 1.26)	0.012	1.29 (1.06, 1.52)	1.24 (1.03, 1.45)	0.239
Trunk, g/cm^2^	0.83 (0.69, 1.00)	0.82 (0.66, 1.00)	0.439	0.90 (0.79, 1.06)	0.91 (0.75, 1.09)	0.742
Ribs, g/cm^2^	0.66 (0.50, 0.87)	0.62 (0.50, 0.88)	0.480	0.75 (0.59, 0.89)	0.74 (0.59, 0.97)	0.797
Pelvis, g/cm^2^	0.94 (0.73, 1.15)	0.88 (0.71, 1.13)	0.130	1.02 (0.83, 1.26)	1.00 (0.81, 1.20)	0.217
Spine, g/cm^2^	0.92 (0.78, 1.20)	0.97 (0.70, 1.11)	0.989	1.04 (0.87, 1.26)	1.08 (0.86, 1.35)	0.450
tBMD, g/cm^2^	1.07 (0.93, 1.20)	0.99 (0.83, 1.20)	0.055	1.16 (1.03, 1.33)	1.12 (0.96, 1.31)	0.143
T-score of tBMD	−0.5 (−2.0, 0.9)	−1.5 (−3.5, 1.1)	0.021	−0.8 (− 2.2, 1.3)	− 0.9 (− 2.9, 1.1)	0.134
Z-score of tBMD	0.1 (− 1.2, 1.2)	− 0.2 (−2.0, 1.4)	0.228	− 0.5 (− 1.5, 1.1)	−0.5 (− 2.5, 1.2)	0.442

### Univariate correlation analysis of CAC score in relation to BMD

**Fig. 3:**
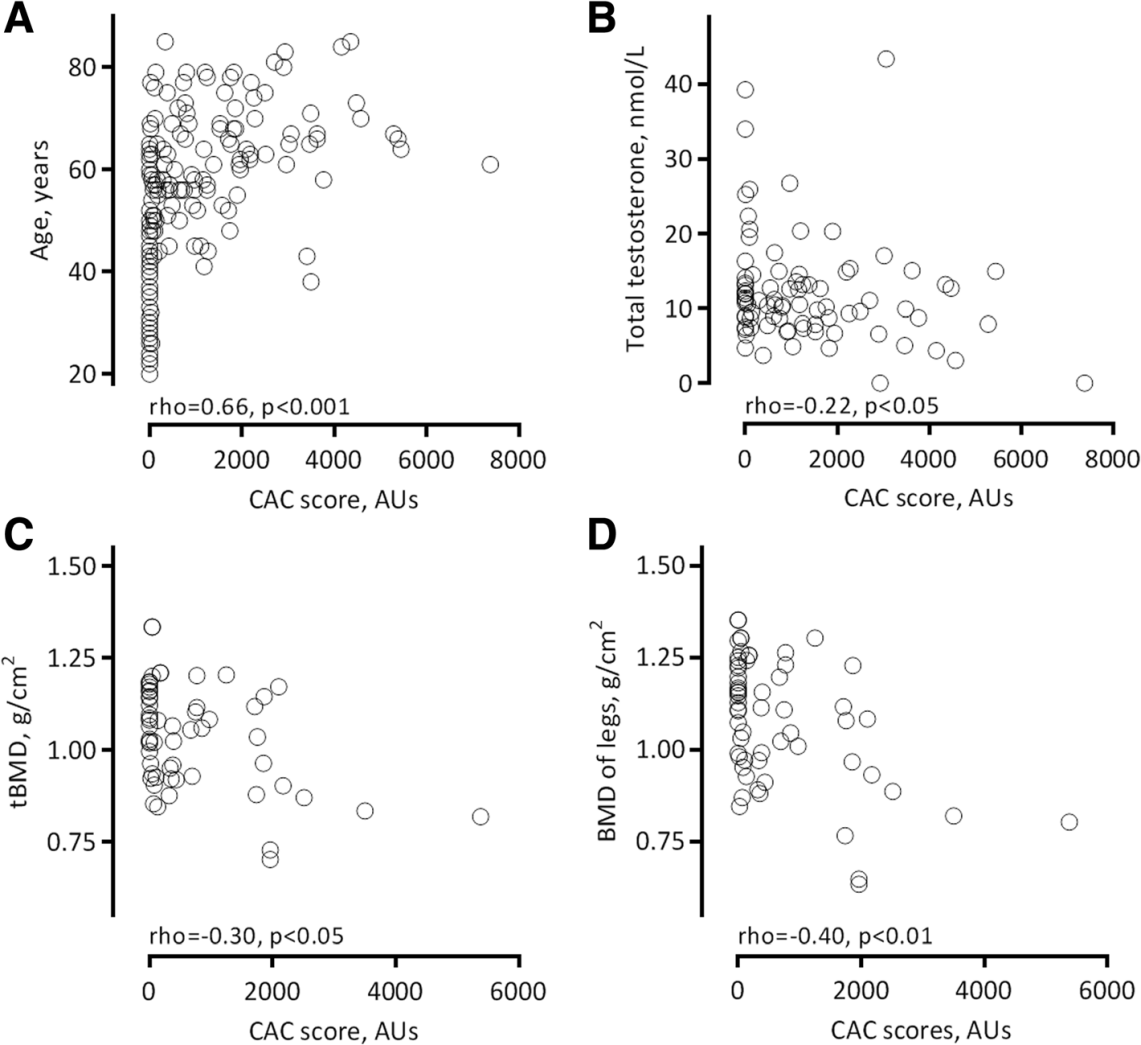
CAC score in ESRD patients associated with: age (*n* = 174, **a**), total testosterone in men (*n* = 95, **b**), and - in women only - with total BMD (*n* = 64, **c**) and BMD at legs (*n* = 64, **d**)

In Spearman rank correlation analysis, CAC score correlated with age (Fig. 3a), diabetes (rho = 0.35, *p* < 0.001), CVD (rho = 0.45, *p* < 0.001), BMI (rho = 0.18, *p* = 0.017), HDL-cholesterol (rho = − 0.18, *p* = 0.019), 1,25-OH vitamin D (rho = − 0.30, *p* < 0.001), albumin (rho = − 0.24, *p* = 0.001) and inflammatory biomarkers: hsCRP (rho = 0.40, *p* < 0.001), IL-6 (rho = 0.51, *p* < 0.001) and TNF (rho = 0.39, *p* < 0.001) significantly. An inverse correlation between CAC score and total testosterone was found in 95 males (Fig. 3b).

Higher CAC score was associated with tBMD (Fig. 3c), T-score and Z-score of tBMD, and sub-regions BMD of arms and legs (Fig. 3d) in females (Table 3). In males, elevated CAC score was only associated with BMD at head (Table 3).

**Table 3:** Spearman’s Rho correlations of CAC score and BMD at sub-regions

	CAC score Rho correlations	
	Female	Male
Head, g/cm^2^	− 0.16	−0.21*
Arms, g/cm^2^	−0.27*	−0.07
Legs, g/cm^2^	−0.40**	−0.07
Trunk, g/cm^2^	−0.16	0.01
Ribs, g/cm^2^	−0.16	−0.01
Pelvis, g/cm^2^	−0.20	−0.08
Spine, g/cm^2^	−0.06	0.14
tBMD, g/cm^2^	−0.30*	−0.08
T-score of tBMD	−0.35**	−0.10
Z-score of tBMD	−0.26*	−0.02

Significant correlations are marked: *P < 0.05, **P < 0.01

### Multivariate generalized linear regression analysis of predictors of CAC score

In multivariate GLM analysis adjusted for age, diabetes and hsCRP found that patients one SD (1057 AUs) higher CAC score was predicted independently by either one SD (0.17 g/cm^2^) lower BMD at the legs (Table 4) or one SD (0.13 g/cm^2^) lower tBMD (Table 5) in female ESRD. No such associations were found in male ESRD patients in whom one SD (1484 AUs) higher CAC score was independently predicted by one SD (16 years) higher age (Table 6 and Table 7).

**Table 4:** Muliple regression for 1-SD (1057 AUs) higher CAC score in 64 female ESRD patients: BMD at legs

Multivariate model (*n* = 64, adjusted r^2^ = 0.17)	Beta	Standard error	*P* value
Higher age (per SD, 16 years)	0.07	0.12	0.58
Higher hsCRP (per SD, 10.5 mmol/L)	0.15	0.12	0.19
Diabetes (yes/no)	0.23	0.27	0.06
Higher BMD at legs (per SD, 0.17 g/cm^2^)	−0.28	0.12	0.02

**Table 5:** Muliple regression for 1-SD (1057 AUs) higher CAC score in 64 female ESRD patients: tBMD

Multivariate model (*n* = 64, adjusted r^2^ = 0.16)	Beta	Standard error	*P* value
Higher age (per SD, 16 years)	0.06	0.12	0.62
Higher hsCRP (per SD, 10.5 mmol/L)	0.16	0.12	0.17
Diabetes (yes/no)	0.27	0.27	0.02
Higher tBMD (per SD, 0.13 g/cm^2^)	−0.27	0.12	0.03

*Abbreviations:* *GLM* generalized linear model, *CAC* coronary artery calcification, *hsCRP* high sensitivity C-reactive protein, *tBMD* total bone mineral density

**Table 6:** Muliple regression for 1-SD (1484 AUs) higher CAC score in 110 male ESRD patients: BMD at legs

Multivariate model (GLM)	Model 1 (*n* = 110, adjusted r^2^ = 0.40)			Model 2 (*n* = 95, adjusted r^2^ = 0.40)		
	Beta	Standard error	*P* value	Beta	Standard error	*P* value
Higher age (per SD, 16 years)	0.61	0.07	< 0.01	0.62	0.07	< 0.01
Higher hsCRP (per SD, 11.8 mmol/L)	0.07	0.07	0.39	0.07	0.07	0.43
Diabetes (yes/no)	0.11	0.15	0.16	0.11	0.17	0.18
Higher BMD at legs (per SD, 0.16 g/cm^2^)	0.12	0.07	0.13	0.16	0.07	0.05
Higher total testoterone (per SD, 7.1 nmol/L)				0.02	0.08	0.78

**Table 7:** Muliple regression for 1-SD (1484 AUs) higher CAC score in 110 male ESRD patients: tBMD

Multivariate model (GLM)	Model 1 (*n* = 110, adjusted r^2^ = 0.40)			Model 2 (*n* = 95, adjusted r^2^ = 0.40)		
	Beta	Standard error	*P* value	Beta	Standard error	*P* value
Higher age (per SD, 16 years)	0.61	0.07	< 0.01	0.61	0.08	< 0.01
Higher hsCRP (per SD, 11.8 mmol/L)	0.06	0.07	0.41	0.06	0.07	0.45
Diabetes (yes/no)	0.10	0.15	0.21	0.10	0.17	0.22
Higher tBMD (per SD, 0.12 g/cm^2^)	0.10	0.07	0.19	0.13	0.07	0.11
Higher total testosterone (per SD, 7.1 nmol/L)				0.03	0.08	0.71

*Abbreviations:* *GLM*, generalized linear model, *CAC* coronary artery calcification, *hsCRP* high sensitivity C-reactive protein, *tBMD* total bone mineral density

When total testosterone in 95 males was added to the model, 1-SD higher BMD at legs (Table 6) but not 1-SD higher tBMD (Table 7) was associated with 1-SD higher CAC score independently.

## Discussion

To the best of our knowledge, this is the first study investigating how sex affects the associations of BMD of different skeletal sub-regions with CAC score in ESRD. Our main observation is that lower tBMD and lower BMD of skeletal sub-regions, in particular at sub-regions of the legs, were associated with increased CAC score independently in females only.

Since several epidemiological studies demonstrated coexistence, but no independent association, of vascular calcification with reduced BMD, it has been proposed that these conditions represent two independent age-related processes [32, 33]. Although they are pathogenically connected [13, 34, 35], the detailed mechanism(s) for the relationship between vascular calcification and BMD are still unclear. Low BMD is associated with an increased risk of CVD [6, 10, 36] and predicts cardiac events and increased mortality in CKD patients and the general population [6–8]. Low BMD and vascular calcification are both common features of the uremic phenotype [37]. Kim et al. [18] found - in a statistical model stratified by gender - when adjusting for age and comorbid conditions, there is no significant association of BMD with CAC. We report that in females, whereas lower BMD, in particular sub-regions of legs, was associated with a higher CAC score independently, even after adjustment for age, diabetes and hsCRP. No such associations were observed in males. Our observation that sex should be taken into consideration when links between bone health and vascular calcification are assessed corresponds with other reports showing associations between lower BMD and presence of CAC in ESRD [6, 9, 10].

It is not known which specific bone location is optimal when studying links between BMD and vascular calcification. It is not evident how one should interpret our observation that tBMD and in particular BMD of legs rather than BMD of other central locations was associated to CAC score. In non-renal patients, lower BMD of the spine, but not the hip [14], was associated with aortic calcification independently. In contrast, Disthabanchong et al. [7] found that in haemodialysis patients, CAC was negatively associated with BMD of hip but not with BMD of lumbar spine. Uyama et al. [35] found an inverse correlation between carotid plaque score and tBMD, but no association with BMD of lumbar spine. Banks et al. [34] found in women that reduced BMD of the proximal femur associated with aortic calcification. Kohno et al. [38] reported in male HD patients association of more bone loss of the ultra-distal radius with higer mortality. Those inconsistent results may be related to differences in population demographics, methods and anatomical sites of measurement of vascular calcification. Nevertheless, in this study, when examining BMD by DXA of different skeletal sub-regions, the correlation between BMD and CAC was more significant in legs than the spine. Since DXA may be influenced by signals from a calcified aorta [7], we presume that the value for spine BMD may be overestimated.

Our report on a significant inverse relationship between BMD and CAC in females only accords with previous studies [39, 40]. Bakhireva et al. [41] reported that higher BMD was associated with reduced CAC of all skeletal sites after adjustment for confounders in women using hormone therapy but not in males, nor in females not on HT. Kim et al. [18] also reported that a significant relationship between BMD and CAC existed only in women. In accordance, Campos-Obando et al. [19] found significant associations between BMD loss and follow-up CAC only in the subgroup of women with lower estradiol levels. Kiel et al. [42] from the Framingham cohort reported a lack of association between BMD loss and aortic calcification in men. Jensky et al. [43] found the association of BMD and CAC to be stronger in women without dyslipidemia in the multi-ethnic study of atherosclerosis cohort. The independent inverse association between BMD and CAC observed in females only suggests the link between arterial calcification and bone mineralization are mediated by sex hormones.

In nondialysis male CKD patients, the reduction in testosterone levels observed with progressive CKD was inversely associated with endothelial dysfunction and exacerbated the risk of future cardiac events [44]. Testosterone concentrations inversely correlate with CVD related and all-caused mortality in CKD patients [45]. Studies investigating possible links of testosterone with vascular calcification have been inconsistent. Whereas two studies show no relationship in males and females [46, 47]; Phillips et al. [48] found in women an inverse relationship between free testosterone and severity of coronary disease. Increasing evidences points towards a protective effect (low testosterone levels associated with high cardiovascular risk) in both females [49–51] and males [52–55]. For the first time, we report on a significant inverse relationship between total testosterone and CAC score among the men. Testosterone has positive effects on endothelium by directly stimulating endothelium-derived nitric oxide [56] and stimulate endothelial progenitor cells, which play a key role in endothelial repair [57]. Furthermore, while testosterone has anabolic effects, including promotion of muscle strength and muscle mass, bone density and maturation, women have much lower levels of testosterone than men. On the other hand, deficiency of estrogen, whose beneficial effects on the coronary arteries have been reported only in women [58], has been proposed to be a common mediator in the emergence of CVD and bone loss in postmenopausal women [59]. The relative contributions of estrogen and testosterone to skeletal homeostasis that may initiate bone loss are still uncertain [60]. It is can be speculated that dysregulation of testosterone and testosterone deficiency may have accounted for the observed sex difference with inverse association of BMD with CAC only in women while in men – when testosterone was added into the model – *higher* BMD associated with higher CAC. This intriguing observation seems to provide further support for sex differentiating links between hormonal status, BMD and vascular calcification.

This study should be interpreted with some limitations. First, no conclusions can be made regarding causality because of the observational design of the study. Second, due to the limited number of participants and the risk of type-2 statistical error, our findings should be interpreted with caution. Third, there are limitations of the methods applied: for measurement of BMD, DXA may not be an ideal method, since it cannot distinguish between bone mineral content and extra-osseous calcifications; and, use of CT for measurement of CAC could not distinguish medial from intimal calcification [15]. Fourth, data on hormone replacement therapy, menopause and sex hormone levels in women were not available.

## Conclusions

In conclusion, lower BMD (tBMD and BMD of sub-regions, in particular the sub-region of legs) was associated with higher CAC scores independently, but only in female ESRD patients. Our main finding that low BMD of legs significantly associates with high CAC scores even when adjusted for age, diabetes and hsCRP should encourage further studies to elucidate the specific mechanisms linking regional differences in bone metabolism and gender differences to vascular calcification.

## Additional file

Additional file 1: **Table S1.** Clinical and biochemical characteristics for the total 174 ESRD patients and for two subgroups based on median total body BMD (tBMD) level. Data presented as median (range of 10th - 90th percentile) or percentage. Abbreviations: BP, blood pressure; HDL, high-density lipoprotein; hsCRP, high sensitivity C-reactive protein; TNF, tumor necrosis factor; IL-6, interleukin-6; PTX, pentraxin; iPTH, intact parathyroid hormone; CAC, coronary artery calcification; tBMD, total bone mineral density. ^a^; *n* = 151, ^b^; *n* = 166, ^c^; *n* = 135; ^d^; *n* = 95; ^e^; *n* = 130, f; *n* = 105. (DOC 52 kb)

## Abbreviations

AUC: Area under the curve
AUs: Agatston units
BMD: Bone mineral density
BMI: Body mass index
BP: Blood pressures
CAC: Coronary artery calcification
CKD: Chronic kidney disease
CKD-MBD: Chronic kidney disease - mineral and bone disorders
CT: Computed tomography
CVD: Cardiovascular disease
DXA: Dual-energy X-ray absorptiometry
ESRD: End-stage renal disease
GE: General Electric
GLM: Generalized linear model
HDL: High density lipoprotein
hsCRP: High-sensitivity C-reactive protein
IL-6: Interleukin-6
iPTH: Intact parathyroid hormone
LD-Rtx: Living donor kidney transplant
PD: Peritoneal dialysis
PTX3: Pentraxin-3
ROC: Receiver operating characteristics
tBMD: Total body BMD
TNF: Tumour necrosis factor
