# Supplementary material for: Differences in association of lower bone mineral density with higher coronary calcification in female and male end-stage renal disease patients
Source: BMC Nephrol. 2019 Feb 18;20:59. doi: 10.1186/s12882-019-1235-z (PMC6380026; doi:10.1186/s12882-019-1235-z)
Supplement: Supplementary file 1 — Table S1. Clinical and biochemical characteristics for the total 174 ESRD patients and for two subgroups based on median total body BMD (tBMD) level. Data presented as median (range of 10th - 90th percentile) or percentage. Abbreviations: BP, blood pressure; HDL, high-density lipoprotein; hsCRP, high sensitivity C-reactive protein; TNF, tumor necrosis factor; IL-6, interleukin-6; PTX, pentraxin; iPTH, intact parathyroid hormone; CAC, coronary artery calcification; tBMD, total bone mineral density. a; n = 151, b; n = 166, c; n = 135; d; n = 95; e; n = 130, f; n = 105. (DOC 52 kb) [file 12882_2019_1235_MOESM1_ESM.doc]

**Supplementary Table 1.** Clinical and biochemical characteristics for the total 174 ESRD patients and for two subgroups based on median total body BMD (tBMD) level.

| **Variables** | **Total patients**  n =174 | **tBMD** |  | **P value** |
| --- | --- | --- | --- | --- |
|  |  | ≤ 1.117 g/cm^2^ (n=87) | > 1.117 g/cm^2^ (n=87) |  |
| **Demography and metabolic biomarkers** | | | | |
| Age, years | 57 (29, 75) | 58 (31, 75) | 53 (26, 74) | 0.102 |
| Male, % | 63 | 49 | 77 | **<0.001** |
| Diabetes, % | 28 | 28 | 29 | 0.866 |
| CVD, % | 22 | 26 | 18 | 0.203 |
| Body mass index, kg/m^2^ | 24.8 (19.9, 30.8) | 23.3 (19.4, 28.7) | 26.3 (21.6, 32.7) | **<0.001** |
| Systolic BP, mmHg | 139 (114, 169) | 140 (115, 167) | 139 (112, 173) | 0.863 |
| Diastolic BP, mmHg | 83 (67, 97) | 82 (67, 99) | 83 (68, 96) | 0.467 |
| Hemoglobin, g/L | 114 (94, 130) | 115 (93, 129) | 111 (96, 130) | 0.067 |
| Triglycerides, mmol/L | 1.6 (0.9, 3.0) | 1.6 (1.0, 2.8) | 1.6 (0.9, 3.3) | 0.732 |
| Cholesterol, mmol/L | 4.7 (3.3, 6.6) | 4.8 (3.6, 6.9) | 4.5 (3.2, 6.3) | 0.076 |
| HDL-cholesterol, mmol/L | 1.2 (0.8, 2.3) | 1.4 (1.0, 2.5) | 1.1 (0.7, 1.7) | **<0.001** |
| Creatinine, µmol/L | 727 (491, 1012) | 671 (469, 928) | 816 (551, 1164) | **<0.001** |
| S-albumin, g/L | 33 (26, 39) | 33 (26, 39) | 33 (28, 39) | 0.597 |
| hsCRP, mg/L | 2.1 (0.4, 18.7) | 1.6 (0.4, 20.5) | 2.4 (0.4, 17.6) | 0.323 |
| TNF, pg/ml ^a^ | 15.3 (9.9, 20.4) | 15.4 (9.7, 20.2) | 15.2 (9.9, 20.7) | 0.898 |
| IL-6, pg/ml ^b^ | 4.5 (0.9, 14.9) | 3.8 (0.9, 15.1) | 4.9 (0.9, 14.9) | 0.822 |
| PTX3 ng/mL ^c^ | 1.7 (0.7, 6.2) | 1.6 (0.7, 5.9) | 1.7 (0.7, 6.8) | 0.547 |
| Total testosterone in male, nmol/L ^d^ | 11.0 (5.8, 20.5) | 10.2 (4.7, 20.4) | 12.1 (6.6,22.9) | 0.134 |
| **Mineral bone disease biomarkers** | | | | |
| iPTH, ng/L | 300 (96, 655) | 305 (118,662) | 270 (86, 664) | 0.433 |
| Calcium, mmol/L | 2.3 (2.0, 2.5) | 2.3 (2.0, 2.5) | 2.3 (2.0, 2.6) | 0.670 |
| Phosphate, mmol/L | 1.8 (1.2, 2.5) | 1.7 (1.1, 2.4) | 2.0 (1.4, 2.6) | 0.005 |
| 1,25-OH vitamin D, nmol/L ^e^ | 13 (9, 28) | 12 (9, 28) | 14 (9, 30) | 0.630 |
| 25-OH vitamin D, ng/L ^f^ | 29 (13, 70) | 29 (16, 75) | 29 (13, 70) | 0.394 |
| CAC score, AUs | 364 (0, 2992) | 435 (0, 2553) | 177 (0, 3457) | 0.280 |

Data presented as median (range of 10^th^ - 90^th^ percentile) or percentage.

Abbreviations: BP, blood pressure; HDL, high-density lipoprotein; hsCRP, high sensitivity C-reactive protein; TNF, tumor necrosis factor; IL-6, interleukin-6; PTX, pentraxin; iPTH, intact parathyroid hormone; CAC, coronary artery calcification; tBMD, total bone mineral density.

^a^; n=151, ^b^; n=166, ^c^; n=135; ^d^; n=95; ^e^; n=130, f; n=105
